# The role of small ruminants in the epidemiology of leptospirosis

**DOI:** 10.1038/s41598-022-05767-x

**Published:** 2022-02-09

**Authors:** Mohammad Rahim Haji Hajikolaei, Sareh Rezaei, Ali Reza Ghadrdan Mashhadi, Masoud Ghorbanpoor

**Affiliations:** 1 Department of Clinical Sciences, Faculty of Veterinary Medicine, Shahid Chamran University of Ahvaz, Ahvaz, Iran; 2 Department of Pathobiology, Faculty of Veterinary Medicine, Shahid Chamran University of Ahvaz, Ahvaz, Iran

**Keywords:** Microbiology, Diseases

## Abstract

Leptospirosis is a common global zoonotic disease of man and all farm animals. Although most leptospiral infections in sheep and goats are asymptomatic, they may play a role in the epidemiology of the disease by the spread of *Leptospira* through the urine. This study was carried out to evaluate the role of sheep and goats in the epidemiology of leptospirosis. Blood and urine samples were taken from 210 goats and 246 sheep. To detect antibodies, sera samples were tested with 8 live serovars of *L. interrogans* (Hardjo, Pomona, Grippotyphosa, Canicola, Ballum, Icterhemorrhagiae, Tarasovi, and Australis) by MAT. Then, urine samples were tested by Nested PCR targeting 16S rRNA gene for detection of pathogenic *Leptospira*. Results of MAT showed that 10.95% of goats and 8.53% of sheep had antibodies against at least one examined serovars. In both species, the highest reacting was *L. i.* Pomona with a rate of 68.18% and 56% in sheep and goats, respectively. Moreover, in PCR, 2 (0.95%) urine samples of goat and 12 (4.87%) urine samples of sheep were positive. All of the MAT positive studied animals were PCR negative and, statistical analysis showed that there was no relationship and agreement between the results of PCR and MAT in sheep (kappa = − 0.07, p > 0.05) and goats (kappa = − 0.02, p > 0.05). Finally, it is concluded that sheep and goats can excrete *L. interrogans* in the urine and thus transmit them to other animals and humans.

## Introduction

Leptospires, spirochetes belonging to the family of leptospiraceae, are the causative agents of leptospirosis which is a common global zoonotic disease of both man and all farm animal species especially in sub-tropical and tropical regions of the world. Currently, over 250 pathogenic serovars and 24 pathogenic serogroups are recognized^1^. Leptospirosis is an emerging infectious disease of humans with a marked increase in the number of cases and frequent outbreaks in South-East Asia and Latin America. Humans are most commonly infected through occupational, recreational, or domestic contact with the urine of carrier animals, either directly or via contaminated water or soil^2–4^.

Although this disease is rare in sheep and goats that good descriptions of the naturally occurring disease in them are lacking^1^, but the clinical signs related to leptospiral infection in these animals are as follows: high fever, abortion, stillbirth, agalactiae, and prenatal death. Lambs and kids especially those in poor condition, are most susceptible and consequence leptospiral infection may manifest fever, haemoglobinuria and jaundice which may result in death^1,5,6^.

Because of the leptospiral fragile nature, the cost and complexity of the isolation method, and the long incubation period, most diagnostic laboratories do not attempt to isolate leptospires. Therefore, diagnosis of leptospiral infection has been based generally on serological methods. Among serological methods developed for the recognition of leptospirosis, the Microscopic Agglutination Test (MAT) is the most commonly used one that provides more information about the serovars in each area^1,7,8^. Unfortunately, it is less useful in the diagnosis of the early stage and chronic disease. In addition, the main challenge of the MAT is the maintenance of live *Leptospira* cultures, which are difficult to grow in a laboratory^8^, and the major concern is the failure of the MAT to differentiate antibody response to natural infection and those after vaccination^1^. The available serological techniques for the diagnosis of leptospirosis have low sensitivity during the early stage of the disease. Therefore, early diagnosis of leptospirosis is important because the severe leptospiral infection can have a fulminant course. Efforts for solving this problem resulted in developing simpler, effective, efficient, and inexpensive diagnostic methods, e.g. polymerase chain reaction (PCR) which has also been used to detect leptospirosis in farm animals. In fact, PCR is faster and more sensitive than conventional methods. PCR assay has been used on various clinical specimens such as urine, blood, semen, and aborted fetus in order to detect leptospiral DNA. Also, PCR may be used for differentiation of pathogenic and saprophytic leptospires^9–17^.

Serological studies have been carried out in sheep and goats and humans in Ahvaz showed that leptospiral infection rate was 14.9%, 10.46%, and 17.7%, respectively^6,18,19^. Based on clinical signs and response to treatment, leptospirosis has been diagnosed in cattle and buffaloes in the hospital of Faculty of Veterinary Medicine in Shahid Chamran University of Ahvaz. Since the source of infection is an infected animal that contaminates pasture, drinking water, and feed by infective urine, aborted fetuses, and uterine discharges, all of the leptospiral serovars are transmitted within and between species in this way^1^. In addition, it should be considered the importance of the zoonotic potential of this bacteria that is transmitted from carrier animals to the humans such as practitioners, farmers, and slaughterhouse workers who present direct contact with those animals and their secretions and excretions especially infected urine^20^. According to the previous studies on leptospiral infection in farm animals and humans, the role of small ruminants in the epidemiology of leptospirosis has been questioned. Therefore, the present study was aimed to determine the role of sheep and goats in the epidemiology of leptospirosis through the detection of seropositive sheep and goats to Leptospira using MAT and infected animals with PCR in urine samples.

## Results

### MAT

According to the results of MAT, 21 (8.53%) sheep and 23 (10.95%) goats had antibodies to one or more serovars (Table 1). Only one goat (4.76%) and two sheep (8.69%) had antibodies to more than one serovar. There was no significant differences (p = 0.428) between these species. *L. interrogans* serovar Pomona was the dominant serovar in sheep (68.2%) and goats (56%), and the other serovars were Icterohaemorrhagiae (18.2%), Hardjo (4.5%), Canicola (4.5%), and Grypothyphosa (4.5%) in sheep and Icterohaemorrhagiae (28%), and Canicola (16%), in goats. With the exception of one sample with a titer level of 200, the other samples had a titer level of 100. However, there were a tendency in adult sheep and goats (≥ 3 years) to be more seropositive than the younger animals, but there was no significant difference between age groups in sheep (p = 0.301) and goats (p = 0.363).

**Table 1:** Comparison of *L. interrogans* infection in sheep and goats with MAT.

Animals	No. positive (%)	No. negative (%)	Total
Sheep	21 (8.53)	225 (91.47)	246
Goats	23 (10.95)	187 (89.05)	210
Total	44 (9.64%)	412 (90.36%)	456

### PCR

**Figure 1:**
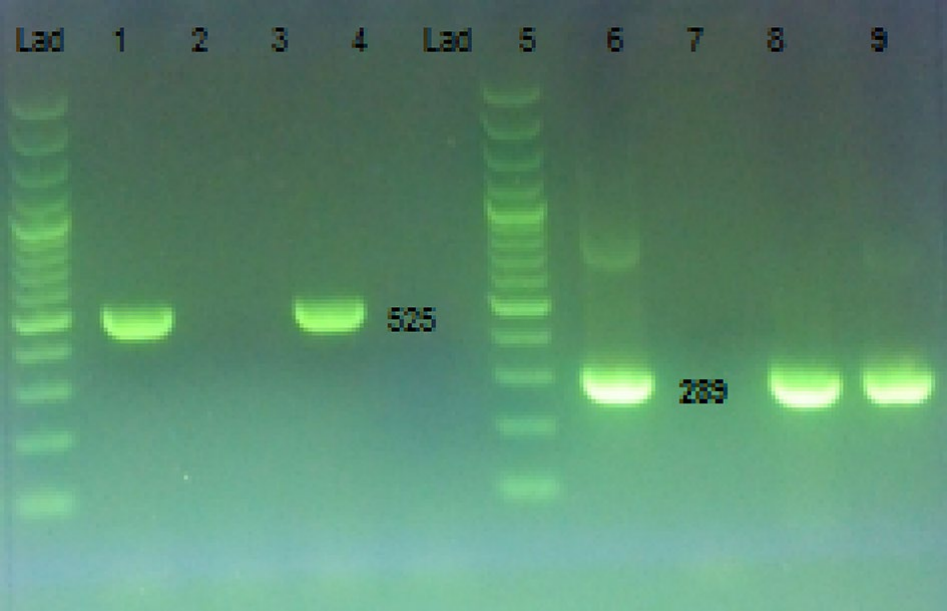
Amplified products from nested PCR of genomic DNA of *Leptospira interrogans* in urine samples. Molecular marker of 100 bp ladder, Lane 1: Positive control 525 bp, Lane 2: Negative control, Lane 3: Positive sample 525 bp, Lane 4: Negative sample, Lane 5: Molecular marker of 100 bp ladder, Lane 6: Positive control 289 bp, Lane 7: Negative control, Lane 8&9: Positive sample 289 bp.

Twelve out of 246 (4.87%) sheep and 2 out of 210 (0.95%) goats were positive in PCR (Fig. 1). Besides, statistical analysis showed that there was no significant difference between sheep and goat (P = 0.26) and also between age groups of sheep and goats (p > 0.05) (Table 2).

**Table 2:** The results of PCR on urine samples of sheep and goats for detection of *L. interrogans* DNA.

Age (year)	Sheep			Goats		
	Positive	Negative	Total	Positive	Negative	Total
< 2	0	10	10	1 (1.69%)	58	59
2	3 (8.1%)	34	37	1 (4.34%)	22	23
3	3 (5%)	57	60	0	37	37
≥ 4	6 (4.32%)	133	139	0	91	91
Total	12 (4.87%)	234	246	2 (0.95%)	208	210

### Relationship between MAT and PCR

Out of 21 sheep and 23 goats that were positive in MAT, none of them was positive in PCR. As a matter of fact, all of the samples that were positive in PCR had no antibodies to *L. interrogans* in MAT. Statistical analysis by McNemar's test showed that there was no correlation between MAT and PCR and Kappa for sheep and goat was − 0.07 and − 0.02, respectively (Tables 3, 4).

**Table 3:** Relationship between the results of MAT and PCR in sheep.

PCR	Positive	Negative	Total
**MAT**			
Positive	0	21	21
Negative	12	213	225
Total	12	234	246
P	> 0.05		
Kappa	− 0.07

**Table 4:** Relationship between the results of MAT and PCR in goats.

PCR	Positive	Negative	Total
**MAT**			
Positive	0	23	23
Negative	2	185	187
Total	2	208	210
P	> 0.05		
Kappa	− 0.02		

## Discussion

Leptospirosis which is caused by different serotypes of the *L. interrogans* is an infectious zoonotic disease and can present in most geographical locations; therefore, information about the serotypes in one region may help to more diagnosis of its epidemiology and pathogenesis characters. The results of the present study showed that 8.53% of sheep and 10.95% of goats had antibodies to one or more serovars *L. interrogans.* In comparison to the previous studies in Ahvaz where the prevalence of *L. interrogans* infection reported 14.9% and 10.46% respectively in sheep and goats^6,18^, it is concluded that the prevalence rate of leptospiral infection in goats has not statistically changed but in sheep, it has declined, though over the years no action has been taken to reduce the dissemination of infection such as vaccination or breeding. Therefore, the causes of this change are not known yet. In contrast to the prevalence rate of infection, the dominant serovar in goats has changed from Grypothyphosa to *Pomona* but in sheep, the dominant serovar was *Pomona* in these two studies. In comparison to this study, leptospiral antibodies were detected by using MAT in 62% of goats and 60.7% of sheep in Tanzania. Goats and sheep which are kept around homesteads had higher leptospiral antibodies prevalence (62%), nearly double of the 38% reported in the same species in humid tropical regions of Tanzania^21^. While, in India, 20.89% of goats^22^, and in the Virgin Islands of the USA, 11.1% of goats and 18.2% of sheep were positive by MAT^23^. Most of the studies on leptospiral infection in the world focused on the serological surveys. The results of which confirm that the prevalence of leptospiral infection in goats and sheep is different not only among the countries but also within the regions of one country. These differences may be the consequence of environmental factors and control efforts^1,24–28^. Because of the slow growth rate of *L. interrogans* and long incubation periods, most studies focus on serology, but some studies have been performed to isolate or diagnose *L. interrogans* by culture or molecular methods such as PCR^9–14,16,17,21,23,29^. Several protocols of PCR, for the detection of leptospiral DNA, have been developed since the 1990s^2^. In fact, PCR is a rapid and sensitive diagnostic technique for the detection or leptospiral DNA in body fluids and tissues and is able to detect up to ten leptospires per milliliter of urine^30^. A variety of primers, some of which are only specific for the genus *Leptospira* and others designated to identify only pathogenic species, are used, These PCR assays do not identify the infecting serovar, although it is possible to identify a specific species by sequencing the PCR amplicons^1^. PCR-based tests have focused on both universal genes present in bacteria, as *gryB*, *rrs* (16S rRNA gene) and *secY*; and surface proteins restricted to *Leptospira,* such as *lipL21, lipL32, lipL41* and *ligB*. The 16S rRNA gene has a good capacity to discriminate between pathogenic, intermediates, and saprophytic leptospires and 24.5% of documented studies have applied it. Although *lipL32* gene is currently the most common target used for *Leptospira* detection, with 48% of studies applying this genetic, but some recent studies still use 16S rRNA gene as a genetic target^31^. According to the literature, the PCR method targeting the 16S ribosomal RNA subunit are the useful diagnostic molecular technique for the direct diagnosis of leptospirosis and is able to detect approximately 10 genome equivalents /mL of whole blood, while the PCR method targeting the LipL32 surface protein gene region is able to detect 100 leptospira/mL of whole blood^32^. So the 16S rRNA-PCR method is more sensitive and more specific than LipL32 PCR technique and in this research, like others^32–34^, the 16S rRNA-PCR method has been used for molecular diagnosis of leptospires in the urine. A disadvantage of PCR is that it can only quantify leptospires, but it cannot identify the specific strain which is important in epidemiological surveillance^8^. In the current study, PCR was used to detect the *L. interrogans* antigen in the urine of both sheep and goats. According to the result of PCR, it was found that 4.87% and 0.95% of the studied sheep and goats was shedding *L. interrogans* from urine and contaminated environment and thus infecting other animals and human being in this way.

In spite of the importance of PCR to detect *L. interrogans* and definitive diagnosis, there is a poor correlation between this method and MAT. Lilenbaum et al. showed that 6 cases of 19 seropositive goats and 6 cases of 40 seropositive sheep were urine PCR-positive^29^. Similarly, Vihol et al., reported there was low concordance between MAT and PCR. Because seroreactivity was noted in 61 goats while leptospiral DNA was detected in 42 goats. Only 18 were positive in both tests and 24 seronegative were positive in PCR^22^. In the study of Cranford et al., 6.3% of sheep and 6.3% of goats were positive by rt-PCR^23^. In the current study, also no correlation between MAT and PCR has been found. All of the goats and sheep that were positive in PCR were seronegative and did not have any antibodies to examined serovars of *L. interrogans* in MAT. Although detection of leptospiral DNA with PCR in urine was found to be highly sensitive, several studies reported a considerable number of seronegative individuals that were *Leptospira* positive by urine PCR, which may be due to the characteristic of *L. interrogans*. This regard, the hosts are divided into two groups, i.e. maintenance and incidental. The first group is characterized by the persistence of the strain in the kidney and sometimes the genital tract with chronic excretion of the pathogen in urine and low antibody response to infection that creates problems in diagnosis; in contrast, the second group is characterized by a short kidney phase and marked antibody response to infection making diagnosis easier^1^. In addition, for many years, small ruminants had been considered as accidental hosts of leptospires, but several studies have shown that leptospiral infection in goats and sheep is common and these species can also act as only maintenance hosts and carriers of leptospires eliminating the agent on the environment for long periods^29,30^. On the other hand, in the serological survey, few serovars are used in MAT. In the current study, only 8 serovars of *L. interrogans* were used. It is supposed that some of the studied sheep and goats may be maintenance hosts for other serovars which not used for MAT. Although, they had no detectable antibodies to these serovars, may excrete from urine that was detectable by PCR. Therefore, the lack of relationship between the results of MAT and PCR can be attributed to the characteristics of *L. interrogans*, low-grade and intermittent leptospiruria, type, and number of serovars are used in MAT. In addition, MAT as a gold standard test is an indirect diagnostic method to detect anti-*Leptospira* antibodies, but PCR is a direct test that allows the identification of leptospiral DNA, therefore, it is expected that there is not much concordance.

## Conclusion

According to the results of the present study, it is concluded that *L. interrogans* is excreted from the urine of sheep and goats although the rate of leptospiruria in these animals was lower than the seroprevalence rate. This confirms the importance of small ruminants as carriers of *L. interrogans* in the epidemiology of leptospirosis. They transmit *L. interrogans* to the other animal species and humans especially those presenting direct contact with the above-mentioned animals and their excretions. On the other hand, for the lack of relationship between two methods, i.e. MAT and PCR, it is recommended that the combination of MAT and PCR be used particularly in epidemiological studies.

## Methods

### Area of the study

This study was carried out in the suburbs of Ahvaz, the center of Khuzestan Province which is located in the Southwest of Iran. Khuzestan Province is located between 48°E and 49.5°E longitudes and 31°N and 32°N latitudes, with an area of 63,213 km^2^ and 27 cities, has the height above sea level between 0 and 3740 m. This province with a climate from arid to humid and its northern parts have cold weather, whereas the southern and central parts have tropical weather^35^.

### Sample size

According to previous serological studies, the mean rate of *L.interrogans* infection in farm animals in this area was reported 34.29%^6,18^. Therefore, the sample size was determined (240 samples), by using the following formula^36^ with 95% confidence level and 6% precision.

$$n=\frac{{\left({Z}_{1-\frac{\alpha }{2}}\right)}^{2}P(1-P)}{{\left(d\right)}^{2}}$$

### Animals and sampling

Blood and urine samples were randomly collected from 246 sheep and 210 goats. Information about each sample, including age (by dental formula), and geographical location were also documented. All of the sheep and goats were female and divided into four age groups; < 2, 2, 3, and ≥ 4 years old. In these age groups, there are 10, 37, 60, and 139 sheep, and 59, 23, 37, and 91 goats, respectively. Sera were stored at − 20 °C until Microscopic Agglutination Test (MAT).

Urine samples were taken by two different methods: in sheep by occluding their nostrils and threatening asphyxia (breathing arrest) and in goats by catheterization^1^. Urine was centrifuged for 10 min at 2500×*g* and sediments were stored at − 20 °C until PCR.

All of the animals were female and at the time of sampling appeared healthy with no clinical signs related to leptospirosis and without any history of vaccination to leptospirosis.

### MAT

The sera were tested by using Microscopic Agglutination Test (MAT) for detection antibodies to *leptospira interrogans*. For this purpose, 8 live serovars of *L.interrogans* (Pomona, Canicola, Hardjo, Ballum, Ictrohemorrhagiae, Grippotyphosa, Tarasovi, and Australis) were used. This test was carried out in leptospiral research laboratory in the Faculty of Veterinary Medicine, University of Tehran according to the methods of OIE. Sera were initially screened at a dilution of 1:100. So that, serum dilution of 1:50 was performed and added to each well of microtitrations plates and then a volume equal of each serovar was added to each well. The microtitrations plates were incubated at 29 °C for 2 h. The plates were examined by dark-field microscopy. Later, the results were considered positive when 50% or more agglutination of leptospires was found^37^. Sera with a positive results were titrated against reacting antigens in serial two-fold dilution from 1:100 to 1:800.

### PCR

DNA from urine samples was extracted according to the method suggested by Burhan et al.^38^. Briefly, 600 µl of 1 mM EDTA (Merck. Germany) was added to 600 µl of urine sediment and after 15 s vortex, the mixture was centrifuged at 13000 rpm for 15 min. The supernatant was discarded and the sediment was suspended in 1 ml of sterile distilled water and after 15 s vortex, centrifugation was repeated as above. Finally, 850 µl of supernatant was discarded and the remaining was vortexed and boiled at 100 °C for 15 min. After that, the mixture was centrifuged at 5000 rpm for 5 min and then 100 µl of supernatant was stored at -20℃ and used as extracted DNA (template) in nested-PCR.

The nested-PCR was carried out by the method of Dinparast et al.^39^ using a gradient thermocycler (Eppendorf, Germany). The targeted gene (16srRNA) was amplified with outer and inner primers under conditions listed in Table 5. In the first reaction, 12.5 μl of 2X mastermix (Ampliqon, Denmark), each containing deoxynucleoside triphosphate, MgCl_2_ and Taq DNA polymerase, 3 μl of template DNA, 1 μl of 10 μM of external forward and reverse primers (Table 5) and 7.5 μl of PCR grade water in the final volume of 25 μl, were mixed and the reaction was carried out as thermal cyclers listed in Table 5. Afterward, the nested reaction was done in a 25 μl of the reaction mixture containing 12.5 μl of mastermix, 1 μl of 10 μM of each internal primer, 9.5 μl PCR water, and 1 μl of product from the first PCR reaction, under the thermal conditions listed in Table 5. Finally, 7 µl of second PCR reaction products were evaluated by electrophoresis in 1.5% agarose gels containing Syber Green (Sinagen, Iran). The gels read under UV light and samples with PCR product of a 289 bp band (Fig. 1) were considered positive.

**Table 5:** Primer sequences and thermal conditions used for molecular detection of *leptospira spp* sheep and goates urine samples.

Gene	Description	Sequence	Size	Cycling conditions	Reference
*16sr RNA*	Forward (outer)	5′-GGCGGCGCGTCTTAA ACATG-3′	525	95 °C—5 min; 30 × (95 °C —30 s, 55 °C—30 s, 72 °C —40 s); 72 °C—5 min	Dinparast et al.^39^
	Reverse (outer)	5′-GTCCGCCTACGCACCCTTTACG-3′			
*16sr RNA*	Forward (inner)	5′-CAAGTCAAGCGGAGTAGCAA-3′	289	95 °C—5 min; 30 × (95 °C—30 s, 58 °C—30 s, 72 °C—30 s); 72 °C—5 min	
	Reverse (inner)	5′-TAACCTGCTGCCTCCCGTA-3′			

### Data analysis

Data were analyzed by using the Chi-Square test, Fisher's exact test, and McNemar's test with a confidence level of 95%, which aimed to detect either differences or correlations between all variables.

### Ethical statement

This manuscript has been approved by the research committee of Shahid Chamran University of Ahvaz and documented by number: 95801106, and all experiments were performed in accordance with the proposal approved by this committee.

### ARRIVE guidelines

This manuscript is reported in accordance with ARRIVE guidelines.
